# Contextual awareness, response and evaluation (CARE) of diabetes in poor urban communities in Ghana: the CARE diabetes project qualitative study protocol

**DOI:** 10.1080/16549716.2024.2364498

**Published:** 2024-07-16

**Authors:** Leonard Baatiema, Daniel Llywelyn Strachan, Lydia Osetohamhen Okoibhole, Irene Akwo Kretchy, Mawuli Kushitor, Raphael Baffour Awuah, Olutobi Adekunle Sanuade, Ernestina Korleki Danyki, Samuel Amon, Kafui Adjaye-Gbewonyo, Haim Yacobi, Megan Vaughan, Ann Blandford, Publa Antwi, Hannah Maria Jennings, Daniel Kojo Arhinful, Ama de-Graft Aikins, Edward Fottrell, the CARE Diabetes Team

**Affiliations:** a Department of Health Policy, Planning and Management, School of Public Health, University of Ghana, Legon, Ghana; b Centre for Tropical Medicine and Global Health Research, Nuffield Department of Medicine, University of Oxford, Oxford, UK; c The Nossal Institute for Global Health, University of Melbourne, Melbourne, Australia; d Institute for Global Health, University College London, London, UK; e Department of Pharmacy Practice and Clinical Pharmacy, School of Pharmacy, University of Ghana, Legon, Ghana; f Department of Health Policy Planning and Management, School of Public Health, University of Health and Allied Sciences, Ho, Ghana; g Regional Institute for Population Studies, University of Ghana, Legon, Ghana; h Vital Strategies, New York, USA; i Department of Population Health Sciences, Division of Health System Innovation and Research, Spencer Fox Eccles School of Medicine at the University of Utah, Salt Lake City, UT, USA; j Center for Social Policy Studies, University of Ghana, Legon, Ghana; k Institute for Lifecourse Development, University of Greenwich, London, UK; l Bartlett Development Planning Unit, University College London, London, UK; m Institute of Advanced Studies, University College London, London, UK; n Department of Computer Science, University College London, London, UK; o Department of Health Sciences, University of York, York, UK; p Hull York Medical School, Heslington, UK; q Department of Epidemiology, Noguchi Memorial Institute for Medical Research, University of Ghana, Legon, Ghana

**Keywords:** Diabetes, urban, Ghana, qualitative, non-communicable diseases

## Abstract

Diabetes remains a major, global clinical and public health threat with consistent rises in prevalence around the world over the past four decades. Two-thirds of the projected increases in global diabetes prevalence to 2045 are expected to come from low- and middle-income countries, including those in sub-Saharan Africa. Ghana is typical of this trend. However, there are gaps in evidence regarding the appropriate development of interventions and well-targeted policies for diabetes prevention and treatment that pay due attention to relevant local conditions and influences. Due consideration to community perspectives of environmental influences on the causes of diabetes, access to appropriate health services and care seeking for diabetes prevention and management is warranted, especially in urban settings. The ‘Contextual Awareness, Response and Evaluation (CARE): Diabetes in Ghana’ project is a mixed methods study in Ga Mashie, Accra. An epidemiological survey is described elsewhere. Six qualitative studies utilising a range of methodologies are proposed in this protocol to generate a contextual understanding of type 2 diabetes mellitus in an urban poor population. They focus on community, care provider, and policy stakeholder perspectives with a focus on food markets and environmental influences, the demand and supply of health services, and the history of the Ga Mashie community and its inhabitants. The results will be shared with the community in Ga Mashie and with health policy stakeholders in Ghana and other settings where the findings may be usefully transferable for the development of community-based interventions for diabetes prevention and control.

## Paper Context

- **Main findings:** Diabetes is a major, global health threat with rises in incidence projected in low- and middle-income countries, including Ghana, yet evidence gaps remain related to the development of contextually appropriate interventions and policies for diabetes prevention and treatment.
- **Added knowledge:** Sitting within a larger, mixed methods study entitled ‘Contextual Awareness, Response and Evaluation (CARE): Diabetes in Ghana’, this study design paper introduces six complementary qualitative studies designed to address this gap in Ga Mashie, Accra.
- **Global health impact for policy and action:** Robust methods to describe diabetes burdens and dissemination of evidence are crucial for health policy and impact.

## Background

Diabetes remains one of the major, global clinical and public health threats with consistent rises in prevalence around the world over the past four decades [1]. In 2017, approximately 451 million adults were living with diabetes worldwide with this figure projected to rise to 693 million by 2045 unless concerted prevention efforts and policy relevant measures are taken [2]. There are three main types of diabetes (type 1 diabetes mellitus, type 2 diabetes mellitus (T2D), and gestational diabetes mellitus); T2D accounts for over 90% of all people with diabetes globally [3]. Living with diabetes increases the risk of mortality by 2–3 times including from other non-communicable diseases (NCDs), particularly cancer, cardiovascular diseases, and chronic kidney diseases [4]. It can also lead to catastrophic financial burdens for individuals, communities, and health systems [5]. The International Diabetes Federation in 2015 estimated that most countries allocate 5–20% of their overall healthcare expenditure to diabetes with global annual expenditure estimated at $673 billion rising to approximately $802 billion by 2040 [6]. Such a level of investment can threaten health system robustness, functionality, and resilience, especially in resource-poor settings [7–9].

Two-thirds of the projected increases in global diabetes prevalence to 2045 are projected to come from low- and middle-income countries, including those in sub-Saharan Africa (SSA) [10,11]. In Ghana, diabetes prevalence is estimated among adults to be at similar levels to other SSA countries though these same estimates suggest the figure would be far higher if accounting for those living without a diagnosis [10,11]. Increases in diabetes prevalence have been attributed to a range of factors such as economic growth, globalisation, rural to urban migration, socio-demographic and economic changes as well as the impact of these factors on traditional diets and behaviours and the risks associated with rising obesity [10–18]. Compounding the negative impact of rising diabetes prevalence is uneven access to high-quality care and low levels of awareness of diabetes and its associated risk factors in the Ghanaian population [11,19–21].

Previous studies have explored the contextual factors associated with the provision of NCD care in Ghana [11,20,22–24]. However, there remains a paucity of evidence to support the development of interventions and well-targeted policies for diabetes prevention and treatment that pay due attention to relevant local conditions and influences [24]. For instance, gaps in evidence remain regarding community understandings of health and disease, including demand for and provision of diabetes-related services both within and outside the formal health sector; the built environment and how it impacts living conditions and health-related decision-making; market forces; socio-demographic factors; religious and ethnic identities, the use of data and potential roles for digital technologies for both individual (self-)management and care provision; gender norms and the policy environment; and how all have evolved over time [9,24]. Addressing these gaps is required to implement the best policy and practices for the prevention and management of diabetes advocated by the WHO [25–28]. Indeed, a focus on the context of implementation has been stressed by several recent implementation science focused papers in sub-Saharan Africa [29–31].

The ‘Contextual Awareness, Response and Evaluation (CARE): Diabetes in Ghana’ project is a mixed methods study in Ga Mashie, Accra, that uses epidemiological methods alongside qualitative approaches to generate a contextual understanding of T2D in an urban poor population. The CARE project builds on the earlier work on T2D in Accra, Ghana, by the Regional Institute for Population Studies (RIPS) Urban Health and Poverty Project [11,24,32,33]. The protocol of the quantitative component, based on a survey, has been described elsewhere [34]. In this paper, the CARE qualitative protocol is described which heeds calls in the literature, notably by the Global Alliance for Chronic Diseases, for adequate contextualisation of NCD scale-up research and the drawing together of a multi-disciplinary team to do so [35].

## The CARE project aim and qualitative objectives

The CARE project aims to generate data to understand the burden, stakeholder narratives, socioecological drivers, consequences, and responses to T2D in Ga Mashie and identify opportunities for community-based interventions for diabetes prevention and control. The current paper describes the methods for the qualitative component of the CARE diabetes project. The aim of the qualitative studies is to understand the context of the research to better interpret the quantitative findings. The areas of context we focus on are the policies and historical, environmental, and healthcare delivery factors that mediate T2D risk and the social norms and experiences that mediate T2D risk prevention, control and care-seeking opportunities and behaviours in the Ga Mashie area of Accra. These define the primary objectives of the CARE diabetes qualitative studies.

**Figure 1.**
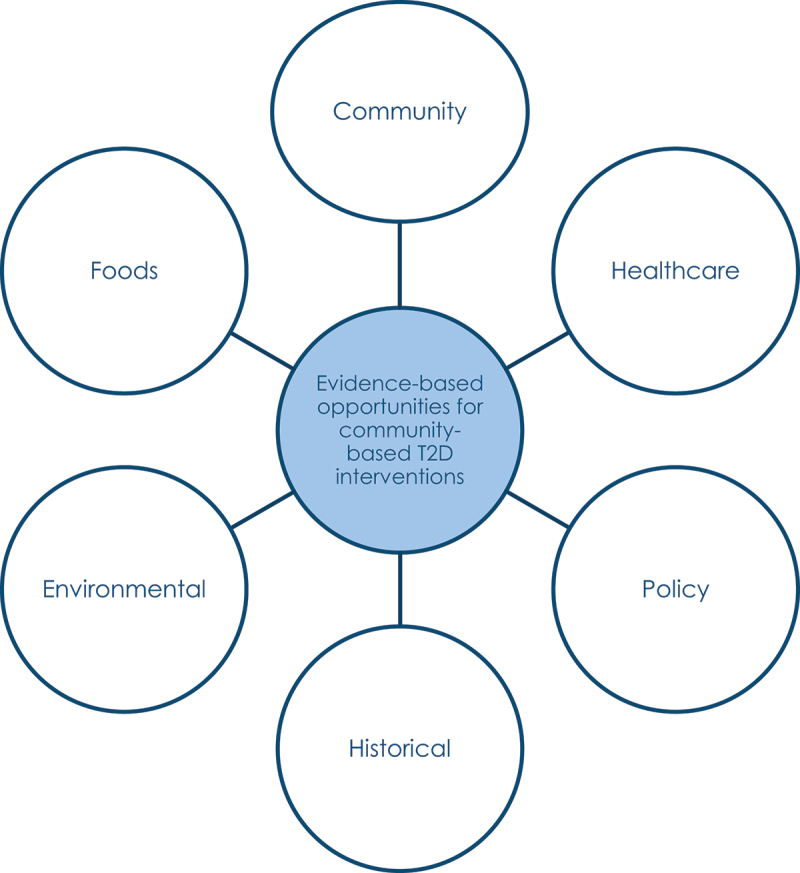
An overview of the CARE diabetes qualitative sub-studies.

Six qualitative studies are planned to complement the quantitative component of the study that will explore the burden of diabetes and its links to socio-economic variables (Figure 1). The content focus, specific objectives and data collection methods for the six qualitative studies are presented in Table 1. The starting point of the qualitative exploration is inductive and exploratory through seeking perspectives of community members and care providers and their choices and opportunities, as well as understanding the policy setting related to diabetes, the local history and the influence of the physical environment (both natural and built).

**Table 1.** Content focus, specific objectives, and data collection methods for the six qualitative studies.

Study	Content focus	Objective	Method
1	Community members	To understand social norms, knowledge, experiences and attitudes regarding risks, prevention, control and care-seeking for T2D, including current accessibility and perceptions of quality of services (formal and informal and use of technology).	Focus group discussions (FGDs)
2	Care providers	To understand T2D care provision and performance priorities (prevention and treatment) including perceived barriers and facilitators of effectiveness and the perceived role of digital and community-based intervention strategies.	In-depth interviews (IDIs) and FGDs
3	Industry, policy, and governance	To understand the policy landscape (policy responses) relating to NCD risk and T2D prevention and treatment through exploration of the implementation of the policies in relation to the WHO Best Buys.[36]	Document review and key informant interviews (KIIs)
4	Historical	To describe local histories of the community, history of health interventions, perceptions on changes in food systems and dietary patterns and practices and how socio-economic activities and political organisation shape the prevention and management of diabetes and health and illness over time in the community.	Document review (archival search), FGDs and IDIs
5	Environmental	To describe and understand the physical attributes of the environment as well as how people make sense and understand how their environment impacts their lifestyle choices.	Cognitive community mapping, spatial mapping and FGDs
6	Healthy and unhealthy food and food choices	To understand the community’s perception of healthy and unhealthy food and the contextual determinants of daily food/meal choices from those living in Ga Mashie.	Photovoice, FGDs

## Methods and analysis

### Study setting

The setting for the study is the Ga Mashie area of Accra, comprised of James Town and Ussher Town. The communities of James Town and Ussher Town are characterised by high population density with a population of around 120,000, low socio-economic status and a built-up environment with poor sanitary conditions and older housing structures. In these communities, monthly household income is low, falling in the lowest of four income classes in urban Accra in a 2010 assessment, and approximately three-quarters of the population have attained education up to Junior High School [32,37]. James Town and Ussher Town are largely indigenous communities inhabited mainly by the Ga-Dangme ethnic group with fishing and petty trading constituting the main economic activities [33].

Contributing to the planning for this protocol and prior to the commencement of data collection, a community-level event was held in Ga Mashie to inform the community, engage with relevant stakeholders such as healthcare workers and the Ga Mashie Development Agency (GAMADA) and ensure community approval of the research. A project inception meeting was hosted at Noguchi Memorial Institute for Medical Research at the University of Ghana in Accra to explain the aims and approach of the project to NCD stakeholders prior to data collection.

### Data collection

A concurrent multi-method, qualitative study design will be employed to meet the study objectives. As outlined in Table 1, data will be gathered from selected individuals and groups within and linked to the community. Data gathering will include focus group discussions (FGDs), in-depth interviews (IDIs), and key informant interviews (KIIs), all based on topic guides tailored to the themes, and other more specialised methods as described below. FGDs and interviews will be conducted by fieldworkers recruited and trained by the project team. They will be conducted in English or Ga as appropriate. Interviews are expected to take between 45 and 75 minutes and FGDs 60 and 90 minutes. Prior to all data collections, participant information sheets will be shared with participants which outline the purpose and approach of the study, the nature of participation and how data will be collected, stored, analysed, and reported as well as contact the details of the researchers.

#### Study 1: Community members

The topic guide will cover themes of perception of diabetes causes and their impact and complications, living with diabetes in the community, management of diabetes in the community (focusing on available services, access to them, and perceptions of quality), and most trusted sources of information (e.g. healthcare professionals, family, friends, online). Other themes are local challenges and enablers of effective, appropriate and valued diabetes care and management (e.g. rising prices), approaches to the monitoring and management of one’s own health (including the use of digital tools), enablers and constrainers of participation in community activities with a view to gauging acceptability and feasibility of a community participatory intervention for diabetes prevention and management, and gendered differences in experience drawing in a similar study conducted in Bangladesh [38].

##### Participants and recruitment

Discrete groups of women and men living with diabetes will be targeted for recruitment as FGD participants. In addition, non-diabetic younger women, older women and younger men, and older men will be recruited. Participants will be adults 18 years and above, both men and women, healthy or diagnosed with T2D. Age delineation for ‘younger’ and ‘older’ participants will be 18–24 years old and 25 years and above. Participants will be recruited by local mobilisers and through local clinics, patient support groups, and across community spaces including households, places of worship, and community meetings within Ga Mashie.

##### Sampling procedure and sample size

To ensure maximum variation in categories and sample size, participants will be recruited through a combination of purposive, convenience, and snowball sampling techniques. The FGDs will be conducted based on gender-based groups as follows: two FGDs each with older women with diabetes, older men with diabetes, older women without diabetes, older men without diabetes, younger women without diabetes, and younger men without diabetes. Each FGD will have six to eight participants. Should opportunities arise for additional FGDs to be conducted within the data collection time frame, they will be taken.

#### Study 2: Care providers

Study 2 will involve a range of formal (defined as orthodox/biomedical health providers within the government sanctioned and funded or private health system) and informal (range of private, commonly fee taking) traditional or alternative health providers. The topic guide will cover themes of awareness and knowledge of T2D, its causes and treatment (NB: knowledge of T2D will only be explored in IDIs), T2D care provision and performance priorities (prevention and treatment), including perceived barriers and facilitators of effectiveness, the perceived role of digital resources, and informed strategies for diabetes care and management, as well as awareness and perspectives on community-based intervention strategies. Again, key themes for this inquiry draw on those generated in a similar study conducted in Bangladesh [38].

##### Participants and recruitment

Study 2 participants will be formal health providers (e.g. medical doctors, physician assistants, nurses of different specialties and responsibilities, community health workers (CHW), community pharmacists, over-the-counter medical assistants and informal health providers (e.g. herbalists, faith healers, and itinerant healthcare providers). Health providers within the formal sector will be recruited through official letters to the Regional Health Directorates and Municipal/District Health Services for the necessary approvals to enter the various health facilities to recruit eligible health workers into the studies. For non-formal care providers, participants will be recruited by local mobilisers through their associations and affiliations in the study communities.

##### Sampling procedure and sample size

A purposive sampling technique will be utilised to recruit relevant healthcare providers from Ussher Polyclinic and pharmacy retail outlets and other health facilities for semi-structured IDIs. Study 2 will comprise at least 32 IDIs. For the FGDs, the study will sample a total of 16–20 formal health providers who will be divided into two groups. Each FGD will include male and female workers and a range of ages and professional experience.

#### Study 3: Industry, policy, and governance

Study 3 will involve stakeholders involved in policy development and influence in Ghana. Efforts will be made to understand the policy landscape (policy responses) in Ghana, relating to NCD risk and T2D prevention and treatment through exploration of the implementation of the policies using the WHO ‘Best Buys’ approach [36]. The topic guide will include questions on a) issues of restriction, regulation, and taxation of harmful products b) the state of implementation of the NCD ‘best buys’, exploring issues around financing and prioritisation of NCDs in policy and care provision, c) barriers to their full implementation, and d) how existing policies are tackling each of the best buys, and the extent of implementation, including barriers and facilitators and recommendations to improve the effectiveness and impact of implementation.

##### Participants and recruitment

Community opinion leaders; policy implementers, policymakers, and developers (from the Ghana Health Service – GHS and the Ministry of Health – MoH); policy influencers (e.g. media outlets and social media, Civil Society Organisations (CSOs), and industry bodies); and relevant NCD stakeholders will be recruited to examine programme and policy prioritisation, financing, and response to NCDs locally. Researchers, the media, CSOs, and donors (influencers) will also be targeted as participants. Recruitment will adopt both formal and informal procedures. For instance, unit heads and administrators will be approached with formal letters of introduction, information sheets, and requests for participants.

##### Sampling procedure and sample size

Study 3 will employ a purposive sampling technique and aim to recruit male and female participants at a range of levels of seniority from across the targeted respondent groups. Thirty participants will be recruited to the KIIs.

#### Study 4: Historical

Study 4 will involve thematic analysis of historical documents as well as the transcripts of IDIs and FGDs with community members and key stakeholders from Ga Mashie. The themes for data gathering and document selection are as follows: the history of health interventions in Ga Mashie (i.e. James Town and Ussher Town) from different perspectives and their success and failures during the pre- and post-colonial periods, with a focus on how experiences and understandings might inform response to future interventions, perceptions on changes in food systems and dietary patterns and practices, market food distribution systems, migration histories of Ga Mashie through discussion of images drawn on the walls of some houses in the two communities, and understanding of how these communities have changed over time, including political and economic changes. Archival work will be conducted using local records with support from local historians.

##### Participants and recruitment

IDIs will be conducted with market women and other key stakeholders involved in food distribution within and outside Ga Mashie, with traditional and religious leaders and community influencers, and with other community members with insights regarding the history of Ga Mashie. Men and women (50 years and above) in James Town and Ussher Town (two male groups and two female groups in each community) will also be recruited to the study as FGD participants. To facilitate recruitment of study participants, traditional leaders will be approached to support identification of people who have in-depth understanding of the history of Ga Mashie generally, as well as the history of health interventions within the community.

##### Sampling procedure and sample size

Study 4 will employ a mix of purposive and snowball sampling techniques to recruit key community leaders and custodians of local history. One GAMADA representative, five traditional and religious leaders, two community opinion leaders and influencers, and ten market men and women will also be approached to participate in the IDIs. Four FGDs with lay adult men and women older than 50 years are planned.

The review of archives will start with exploration based on the same themes.

#### Study 5: Environmental

Study 5 will meet its objectives through mapping processes and community reflection on perceptions of human interaction with the physical environment. The study area (built environment) will be mapped for the risk factors of diabetes (including food, alcohol, and physical activity spaces). Two mapping approaches will be used: spatial mapping and cognitive community mapping [39]. Objective spatial mapping will focus on healthcare facilities and the digital landscape to describe and analyse spatial and environmental factors influencing diabetes risk (e.g. walkability, food access, use of public spaces, digital access). FGDs and cognitive maps will provide sociocultural and historical dimensions to the physical space. The shared experience of the community environmental space may provide perspectives on healthy and unhealthy spaces and perceived structural and social barriers to physical activity, healthier diets, and reliable health information.

##### Participants and recruitment

Participants for both the cognitive mapping and the FGDs must be permanent residents of Ga Mashie and be more than 25 years old to align the data collection with the quantitative protocol of the CARE diabetes project [34]. Recruitment will be based on location (James Town and Ussher Town), primarily because the contexts are different and may present different risk profiles for diabetes, as well as gender, age, and occupation. For the age selection, participants 35 years and above will be considered as an older FGD group and those below 35 years as a younger group. To explore the influence of the environment on the risk of diabetes, market women, fishermen, butchers, and food sellers will be recruited as participants for both cognitive mapping and the FGDs with six to eight participants in each. Study 5 will aim to conduct nine FGDs.

##### Data collection

In preparation and in order to generate the spatial maps, researchers will identify potential risk factors and upstream determinants of diabetes such as food, alcohol, playgrounds, recreational areas, and physical activity spaces as well as health facilities [40]. This will be accomplished by generating a community street map of the area from Google Maps^TM^. The Google Street Maps will aid in delineating the community boundary and help plan the field work street by street. By means of handheld GPS tools and tablets, every food item sold in the community will be geolocated. The aim is to build a complete census of street foods, either cooked or uncooked, by location, type of food, time of day, and type of facility food was sold in. Alcohol, recreational activities, beverages, and physical activity spaces will all be mapped in this way to ensure an accurate representation of the community in terms of how diabetes risk factors are generated.

A cognitive community mapping approach will be utilised to generate maps reflective of community perceptions of the local environment and how they link it to the risk of diabetes in the study communities. Cognitive maps will be generated within FGDs where community members will collaboratively generate a visual representation of the community. The data generated in the physical form of a cognitive map and the subsequent discussion of community observations and interpretations of their environment will be analysed in conjunction with the detailed objective maps described above.

#### Study 6: Food and food choices

Study 6 will exploit the community participatory approach of photovoice that utilises images selected by research participants to stimulate discussion around local perspectives and priorities [41]. This approach will be used to understand the community’s perception of healthy and unhealthy food and the contextual determinants of daily food/meal choices for those living in Ga Mashie. It invites participants to use photography to tell stories and to provide a narrative context about their experiences while prompted by the images they have chosen to take, share, and discuss within an FGD [41].

##### Participants and recruitment

The inclusion criteria for Study 6 are adults aged 18 and over, both men and women, living in Ga Mashie, and with unknown diabetic status. They must have access to a photo-taking device.

##### Sampling procedure and sample size

Participants will be recruited using purposive sampling, using existing networks in Ga Mashie. The aim is to hold at least two FGDs with four to six participants per group and to recruit community members from both James Town and Ussher Town.

##### Data collection

Prior to data collection, a workshop with participants will introduce the photovoice approach, photography techniques, and etiquette including privacy and provide examples. It will be explained to participants that they should take photographs of food they consider to be ‘healthy’ or ‘unhealthy’ and that they eat or see being sold in Ga Mashie. Participants will be given up to a week to take photographs and requested to send them to the facilitator prior to the FGDs. Participants will be asked to highlight one to two pictures of ‘healthy’ and ‘unhealthy’ food with the understanding that they will be discussed in the FGD.

The research team will print participants’ photographs ahead of the FGD. Participants will be invited to describe, in front of the group, the photographs they took and selected and explain why they considered them examples of healthy or unhealthy foods. The FGDs will be conducted in a mix of English and Ga languages. A topic guide with questions specific to each of the five criteria of the SHOWeD method [37] will be pre-tested and refined for use in the FGDs. The original SHOWeD criteria and proposed questions appear in Appendix 1.

### Data analysis

All KIIs, IDIs, and FGDs will be digitally recorded and transcribed verbatim by the trained fieldworkers who conducted the interviews and/or facilitated the discussions. All interviews conducted in the local languages (Ga and Twi) will simultaneously be translated with transcription from Ga to the English Language. Transcripts from all the interviews will be analysed thematically with the key themes of the topic guides forming the first draft of the coding frame for each study and emergent themes added through analysis of the data. The analysis will iterate between the data and the initial topic guide to assess whether the *a priori* themes were in the data and whether additional themes need to be added to the coding frame [42]. All transcripts will be imported into QSR NVivo 11 software to facilitate data coding, analysis, and reporting. For Studies 3 and 4, thematic analysis will be conducted on policy documents and historical records, respectively, with the key content focus areas of each study forming the initial analysis framework. For the spatial awareness element of Study 5, individual-level spatial attributes will be measured. Group and community-level attributes will also be examined. For the photovoice FGDs in Study 6, thematic analysis of the focus group transcripts will be conducted with reference to the photographs taken by participants.

The results of all studies will be brought together in a cross-study workshop where key findings from the qualitative and quantitative studies will be synthesised and researchers who participated in the design and implementation of all qualitative and quantitative studies will share and discuss the data collected and emergent findings. The key emphasis of the synthesis will be to address the project objectives of understanding the burden, narratives, socioecological drivers, consequences, and responses to T2D in Ga Mashie, adopting a multidisciplinary inquiry lens to the context, and identifying opportunities for community-based interventions for diabetes prevention and control.

## Trustworthiness and rigour

To ensure the rigour and trustworthiness of the data, multiple approaches have and will be employed. First, a standardised guideline for reporting qualitative studies (the 32-item checklist for interviews and focus groups of the Consolidated Criteria for Reporting Qualitative research) was adopted to guide the design and overall protocol development [43]. All KIIs and FGDs will be digitally recorded and transcribed verbatim, and fieldworkers trained and tasked to take field notes to aid analysis. Both approaches add to the integrity and rigour of the data collection. Member cross-checking of randomly selected transcripts, with constant comparison with the audio data, will be performed on transcripts to be reflective of what respondents communicated. Several members of the multi-disciplinary research team will be involved in data coding of KII and FGD transcripts to minimise bias. Data analysis will be conducted by researchers from different disciplines with the resulting triangulation of research findings aiding rigour and interpretative trustworthiness.

## Patient and public involvement

The CARE diabetes study was developed in collaboration with relevant key stakeholders in the prevention and treatment of NCDs in Ga Mashie, Accra, Ghana. This approach was adopted to ensure community and stakeholder participation and ownership of the project and to align with the National Institute for Health Research recommendation to involve patients and the public/communities in the co-creation of knowledge through research. It was also done to ensure communities and the public are actively involved in every stage of the research process. Institutional support letters were provided by relevant stakeholders from the Ghana Health Service, Food and Drugs Authority, Ghana NCDs Alliance, the Accra Metropolitan Assembly, and GAMADA. These support letters highlighted the relevance and feasibility of this study in the study context. Prior research activities by some members of the research team have involved the collection and analysis of data from people living with diabetes and NCDs, from community leaders, and other stakeholders in Ga Mashie [11,24,33,44]. These data have underpinned the conceptualisation and development of the research aim and objectives of the CARE diabetes study. The project team will be led into the data collection by team members who have experience in community engagement and data collection in Ga Mashie complemented by topic experts with expertise in social psychology and health systems (Studies 1 and 2), policy analysis (Study 3), historical analysis (Study 4), spatial and cognitive mapping (Study 5), and photovoice (Study 6). The project team will continue to engage with community stakeholders including community representatives through the study including when refining study conclusions and when disseminating the findings.

## Ethical approval for the research

This study has received approval from the Ghana Health Service Human Research Ethics Committee (Protocol ID NO: GHS-ERC 017/02/22) and the University College London Research Ethics Committee (Study ID # 21541/001). The protocol was also reviewed by the Noguchi Memorial Institute for Medical Research Institutional Review Board (NMIMR-IRB CPN 060/21–22 IORG 000908). The informed consent of all participants who have volunteered to join in the research will be obtained and recorded prior to their participation in the study. All interviews (audio recordings and transcripts) will be stored on a password-protected data stick (encrypted USB stick). All data will be anonymised. For example, selected quotations from the interviews will be used to highlight or illustrate a key finding and, in this process, participants’ identities will be anonymised. All identifiable participant information will be assigned pseudonyms. The researchers will regularly monitor and check participants’ willingness to continue with the study.

## Dissemination

This study has a multifaceted dissemination plan with the aim of informing the development of community-based interventions for diabetes prevention and control in Ghana and other settings where the findings may be applied. At the completion of the study, community-level dissemination meetings in James Town and Ussher Town and a dissemination event in Accra with governmental, non-governmental, and academic stakeholders will be organised. Findings from the research will be developed into policy briefs highlighting key recommendations for T2D risk factors, prevention, and well-targeted treatment and policies within the context of urban poor communities. The results will also be disseminated in manuscripts published in open-access, academic journals. Findings will be presented at international and national global health conferences and meetings and shared with relevant CSOs, healthcare professionals, researchers, informal caregiver and patient associations, and policy stakeholders.
